# Genomic amplification of *BCR/ABL1 *and a region downstream of *ABL1 *in chronic myeloid leukaemia: a FISH mapping study of CML patients and cell lines

**DOI:** 10.1186/1755-8166-3-15

**Published:** 2010-09-01

**Authors:** Anna Virgili, Elisabeth P Nacheva

**Affiliations:** 1 Academic Haematology, University College London Cancer Institute, Royal Free Campus, Rowland Hill Street, London, NW3 2PF, UK

## Abstract

**Background:**

Chronic myeloid leukaemia (CML) is characterized by the expression of the *BCR/ABL1 *fusion gene, a constitutively activated tyrosine kinase that commonly results from the formation of the Philadelphia (Ph) chromosome after a t(9;22)(q34;q11) or variant rearrangement. The duplication of the Ph chromosome is a recurring abnormality acquired during disease progression, whereas intrachromosomal amplification of *BCR/ABL1 *is a rare phenomenon and has been associated with imatinib therapy resistance. Archival bone marrow chromosome suspensions from 19 CML patients known to carry more than 1 copy of *BCR/ABL1 *and 10 CML cell lines were analyzed by fluorescent in situ hybridization with a panel of probes from 9q34.1-qter to investigate whether they carried two identical copies of the Ph chromosome or, instead, one or both Ph contained cryptic imbalances of some regions.

**Results:**

A duplication of the entire Ph chromosome with no further events involving the derivative 22 was found in 12 patients. In contrast, a sideline with either 1 or 2 isochromosomes of the Ph chromosome was identified in 6 patients but none of the cell lines. In one of the patients a translocation between the distal end of one arm of the isoderivative chromosome 22 and a third chromosome was revealed. 2 patients were found to carry marker structures harbouring high copy number gains of *BCR/ABL1 *fusion along with a variable part of 9q34 region downstream of *ABL1 *breakpoint, similarly to the markers present in the imatinib resistant cell line K562. We identified the following regions of amplification: 9q34.1 → q34.2 and 9q34.1 → qter, with a common minimum amplified region of 682 Kb. One of the patients had 5 *BCR/ABL1 *positive clones with variable level of 9q34 amplifications on a variety of structures, from an isoderivative 22 to tandem duplications.

**Conclusions:**

These data confirm that the intrachromosomal genomic amplification of *BCR/ABL1 *that occurs in some CML patients during disease progression also involves amplification of 9q34 gene-rich sequences downstream of *ABL1 *breakpoint. The variety of rearrangements identified in this relatively small cohort demonstrates that the Ph chromosome is not a stable structure but prone to further rearrangements during disease progression.

## Background

Chronic myeloid leukaemia (CML) is a malignant pluripotent haematopoietic stem cell disease characterized by the expression of the *BCR/ABL1 *fusion gene, a constitutively activated tyrosine kinase which commonly results from the formation of the Philadelphia chromosome (Ph) after a t(9;22)(q34;q11) or related variant rearrangement [1]. Amplification of *BCR/ABL1*, as well as mutations of the fusion gene, has been shown to be associated with clinical resistance to imatinib therapy [2,3]. The blast phase of CML is characterized by acquisition of new cytogenetic abnormalities in 80% of the patients, the most common being trisomy 8, duplication of the Ph chromosome and isochromosome 17 [4]. The additional Ph chromosome houses a second copy of the *BCR/ABL1 *fusion resulting in genomic amplification of the chimeric gene. In addition, extra copies of the fusion gene have also been described housed by isochromosomes derived from the Ph chromosome or marker structures containing tandem duplications of *BCR/ABL1 *fusion [5-8]. Moreover, a study using bacterial artificial chromosome (BAC) array comparative genomic hybridization on CML cell lines and patients [9] revealed unexpected gains of the 9q34 region affecting *BCR/ABL1 *fusion and part of the sequences telomeric to the fusion gene, which could not be explained as due to a simple duplication of the Ph chromosome.

In the present study, archival fixed bone marrow chromosome suspensions from 19 CML patients and 10 CML cell lines known to carry more than 1 copy of *BCR/ABL1 *fusion were analyzed by fluorescent *in situ *hybridization (FISH) in order to investigate whether CML patients with a double Ph have two exact copies of the Ph chromosome or there are instead cryptic chromosomal rearrangements involving *BCR/ABL1 *fusion gene amplification as found in cell lines [9]. Using a panel of BAC probes selected from the long arm of chromosome 9, we screened for and mapped any rearrangements of the Ph chromosome involving gains of the fusion gene. Apart from the duplication of the entire Ph chromosome, we identified in six patients the presence of one or two isoderivative chromosome 22 [ider(22)t(9;22)]. In two patients we detected marker structures harbouring high copy number gains of *BCR/ABL1 *fusion along with a variable part of the 9q34.12-qter region downstream of *ABL1 *breakpoint, similarly to the marker structures present in the imatinib resistant cell line K562. One of the patients showed five *BCR/ABL1 *positive clones with variable level of 9q34.12-qter amplifications on a variety of structures, from an ider(22)t(9;22) to tandem duplications.

## Results

**Figure 1:**
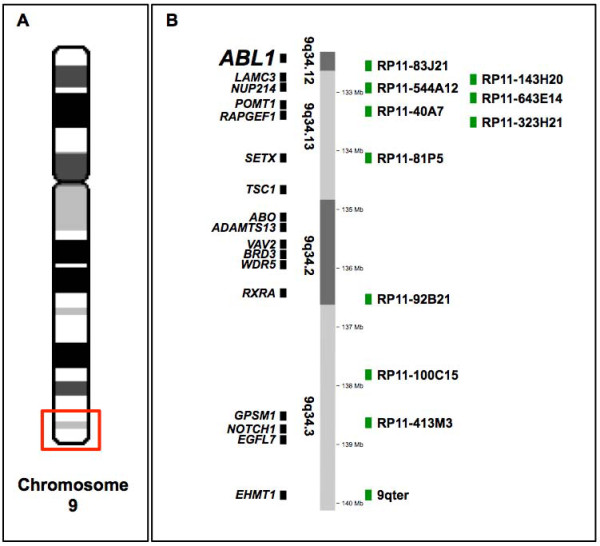
**Schematic representation of the 9q34 region showing the position of the BAC probes**. **(A) **Ideogram of chromosome 9 highlighting in red the 9q34 region that has been analyzed in this study. **(B) **Detail of the 9q34 region indicating the relative position of relevant genes (left) and the BAC probes used in this study (right).

The cytogenetic characteristics of the patients are presented in Table 1, while a schematic representation of the 9q34 region indicating the relative position of the BAC probes used for this study, as well as the relevant genes, is shown in Figure 1. Initial FISH analysis with the probes RP11-83J21 (band 9q34.12) and RP11-323H21 (band 9q34.13) on metaphase cells identified diverse cell populations based on the presence or absence of a typical Ph chromosome: cells without a t(9;22)(q34;q11); with one or two copies of the Ph chromosome; with one or two isoderivative chromosome 22; and with marker chromosomes derived from the Ph (see Table 2). FISH confirmed the presence of two classical Ph chromosomes in a subset of cells of all patients but three (no. 11, 14, 18), one of which had indeed a variant translocation involving the chromosomes 1, 9 and 22 with the *BCR/ABL1 *fusion located on two identical markers der(1)t(1;22;9)(q21;q11;q34), mimicking a duplication of the Ph chromosome. The additional copy of the Ph chromosome was the only rearrangement of the Ph that was detected in twelve patients (no. 1-12). In this group, patient no. 8 harboured a deletion of the normal *ABL1 *gene on the non-translocated homologue of chromosome 9 in Ph positive cells, while patients no. 9, 10 and 11 had variant translocations involving chromosomes 17, 19 and 1, respectively.

**Table 1:** Characteristics of the CML patient's samples

ID	Stage	Karyotype	*BCR/ABL1 *D-FISH (Interphase FISH)
1	BC	46,XY,t(9;22)(q34;q11)[3]/47,idem,+der(22)t(9;22)(q34;q11)[7]	NA

2	CP	46,XY,t(9;22)(q34;q11)[7]/47,idem,+der(22)t(9;22)(q34;q11)[7]	NA

3	NA	NA	NA

4	NA	NA	NA

5	NA	NA	NA

6	NA	NA	NA

7	NA	46,XY,t(9;22)(q34;q11)/47,XY,idem,der(22)t(9,22)(q34;q11)	NA

8	P- SCT	49,XY,+8,del(9)(q31?q34),t(9;22)(q34;q11),add(21)(q22),+add(21)(q22),+der(22)t(9;22)(q34;q11)[8]/46,XX[3]	2R2G[87]/1G3F[13]^1^

9	BC	46,XY,t(17;22)(q23;q11)t(9;22)(q34;q11)[8]/47,idem,+der(22)t(9;22)(q34;q11)[2]/48-50,idem,+Y,+8,+10,+der(22)t(9;22)(q34;q11)[cp3]	NA

10	NA	NA	NA

11	R	47,XY,t(1;22;9)(q21;q11;q34),add(12)(q24),+der(22)t(1;22;9)t(22,?22)(q11;q11)[10]	1R2G1F[11]/1R2G2F[63]/2R2G[26]^2^

12	BC	46,XY[1]/48-49,XY,t(9;22)(q34;q11),+der(22),+8,+8,+8,+8,t(20;21)(q12;q22)[19]inc	2R2G[210]/1R1G3F[17]

13	CP	46,XY,t(9;22)(q34;q11.2)[12]/47,idem,+der(22)t(9;22)[3]	2R2G[34]/1R1G2F[6]/1G2F[58]/1G3F[6]^1^

14	P	NA	Metaphase FISH:2R2G[12]/1R1G2F[5]/1R1 G3F[3]

15	AP	46,XY,t(9;22)(q34;q11)[1]/46,idem,der(19)t(?17;19)(q1?1;p13)[4]/47,idem,+der(22)t(9;22)(q34;q11)[5]	1R1G2F/1R1G3F

16	AP	46,XY,t(9;22)(q34;q11)[7]/55,idem,+8,+8,+14,+18,+18,+19,+21,+der(22)t(9;22)(q34;q11)[63]/46,XY[1]	NA

17	BC- BMT	?47-53,XY,t(9;22)(q34;q11),+der(22)t(9;22)(q34;q11),+1-7 mar[cp11]	Metaphase FISH: 2R2G[3]/2R1G1F[8]/2R1 G2F[9]^3^

18	R	NA	2R2G[67]/1R1G5F[33]

19	BC	46,XY,t(9;22;17;11)(q34;q11.2;q11.2;q13)[2]/46,idem,add(5)(q?)[33]/47,idem,+der(22)t(9;22)(q34;q11)[?]	NA

^1 ^Deletion on the non-translocated homologue of chromosome 9.^2 ^*BCR/ABL1 *fusion on der(1).^3^ Partial deletion of *ABL1*/BCR at der(9). Abbreviations: AP, accelerated phase; BC, blast crisis; BC-BMT, blast crisis post sex mismatch bone marrow transplant; CP, chronic phase; NA, not available; P, presentation; P-SCT, persistent post sex mismatch stem cell transplant; R, relapse.

**Table 2:** Summary of the FISH screening results with the probes RP11-83J21 (band 9q34.12) and RP11-323H21 (band 9q34.13)

ID	Cell clones identified by metaphase FISH analysis		Isoderivative Ph?	**High copy number gains in markers?**
1	i.	t(9;22)[1]	No	No
	ii.	t(9;22),+der(22)[19]		

2	i.	t(9;22)[18]	No	No
	ii.	t(9;22),+der(22)[2]		

3	i.	t(9;22),+der(22)[20]	No	No

4	i.	t(9;22),+der(22)[20]	No	No

5	i.	t(9;22),+der(22)[20]	No	No

6	i.	t(9;22)[7]	No	No
	ii.	t(9;22),+der(22)[13]		

7	i.	t(9;22)[10]	No	No
	ii.	t(9;22),+der(22)[10]		

8	i.	No t(9;22)[12]	No	No
	ii.	t(9;22),del(9),+der(22)[8]^1^		

9	i.	t(9;22)[17]	No	No
	ii.	t(9;22),+der(22)[3]		

10	i.	No t(9;22)[6]	No	No
	ii.	t(9;22)[1]		
	iii.	t(9;22),+der(22)[13]		

11	i.	t(1;22;9)[1]	No	No
	ii.	t(1;22;9),+der(22)t(1;22;9)[19]^2^		

12	i.	t(9;22),+der(22)[6]^3^	No	No

13	i.	t(9;22)[6]	Yes	No
	ii.	t(9;22),del(9) [12]^1^		
	iii.	t(9;22),del(9),+der(22)[2]^1^		
	iv.	t(9;22),del(9),ider(22)[1]^1^		

14	i.	No t(9;22)[10]	Yes	No
	ii.	t(9;22)[4]		
	iii.	t(9;22),ider(22)[1]		

15	i.	t(9;22)[4]	Yes	No
	ii.	t(9;22),+der(22)[12]		
	iii.	t(9;22),ider(22)[1]		

16	i.	t(9;22)[8]	Yes	No
	ii.	t(9;22),+der(22)[10]		
	iii.	t(9;22),ider(22),+ider(22)[1]		

17	i.	No t(9;22)[7]	Yes	No
	ii.	t(9;22)[3]		
	iii.	t(9;22),+der(22)[8]		
	iv.	t(9;22),ider(22)[1]^4^		

18	i.	No t(9;22)[13]	No	Yes
	ii.	t(9;22),marker[1]		

19	i.	t(9;22)[1]	Yes	Yes
	ii.	t(9;22),+der(22)[15]		
	iii.	t(9;22),ider(22),marker[2]		
	iv.	t(9;22),marker[8]		
	v.	t(9;22),marker,marker[8]		

^1^Deletion on the non-translocated homologue of chromosome 9.^2 ^*BCR/ABL1 *fusion on derivative 1.^3 ^FISH analysis on interphase cells showed a ratio of normal cells versus *BCR/ABL1 *positive cells (with an extra fusion signal) of 95% vs 5%.^4 ^Further translocation of the isoderivative 22 with a marker chromosome.

In addition, a subset of cells from seven patients tested positive for other gains of *BCR/ABL1 *fusion apart from the presence of a second Ph chromosome. Two types of chromosome abnormalities were found in these cases: gains of the Ph chromosome taking the form of an ider(22)t(9;22)(q34;q11) chromosome were detected in six patients, while high copy number gains of only part of the 9q34.1-qter region harboured by marker structures derived from the Ph chromosome were found in two patients (see Additional file 1). Remarkably, one patient (no. 19) displayed five different *BCR/ABL1 *fusion positive cell clones with different degree of amplification, ranging from an ider(22)t(9;22)(q34;q11) to markers with tandem duplications, and was therefore included in both groups.

### Metaphase FISH identifies cell populations with an isoderivative chromosome 22, ider(22)t(9;22)(q34;q11), in six patients

**Figure 2:**
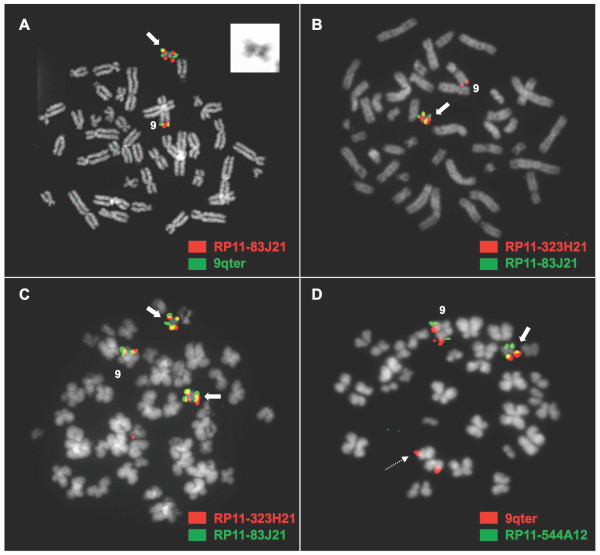
**Gains of the *BCR/ABL1 *fusion taking the form of an ider(22)t(9;22)(q34;q11)**. **(A) **A representative metaphase cell with co-hybridization of FISH probes RP11-83J21 (3' *ABL1*; signal in red) and 9qter (in green) as seen in patients no 14-15, showing a duplicated fusion signal on the isoderivative 22 (block arrow) and a third fusion signal at 9q34 of the unrearranged homologue of chromosome 9. Top right, close-up of the ider(22) DAPI banding. **(B) **A representative metaphase cell from patient no 13 with co-hybridization of FISH probes RP11-83J21 (in green) and RP11-323H21 (5'*RAPGEF1*; in red) showing a duplicated fusion signal on the isoderivative 22 (block arrow). A deletion of the homologue of chromosome 9 not involved in the t(9;22) is detected by the hybridization of only the red signal from RP11-323H21. **(C) **A representative metaphase cell from patient no 16 with co-hybridization of FISH probes RP11-83J21 (in green) and RP11-323H21 (in red) showing the presence of four fusion signals on two isoderivative 22 chromosomes (block arrows) and a fifth fusion signal on the normal homologue of chromosome 9. **(D) **A representative metaphase cell in patient no 17 with co-hybridization of FISH probes RP11-544A12 (*NUP214*; in green) and 9qter (in red), showing a normal 9 homologue and unmasking a cryptic translocation between the ider(22) (block arrow) and a marker chromosome (dashed arrow).

In six patients, a 5-7% of the analyzed metaphase cells carried an isoderivative chromosome 22 replacing the classical Ph chromosome (Figure 2). Five of these patients (no. 13-15, 17, 19) had one ider(22)t(9;22)(q34;q11) effectively representing two copies of a duplicated Ph chromosome, but the sixth patient (no. 16) had two ider(22)t(9;22)(q34;q11) resulting in the presence of four copies of *BCR/ABL1 *fusion in the gene. All six patients had a subclone of cells with only one Ph chromosome; two patients had a Ph negative subclone of cells; and all but one (no. 14) had a subclone with two Ph chromosomes. FISH with a commercial 9q sub-telomeric probe confirmed the identity of the ider(22)t(9;22)(q34;q11) hybridizing at both ends of the isochromosome in all patients but one (no. 17), in whom the 9q sub-telomeric probe unmasked a translocation between the ider(22)t(9;22)(q34;q11) and a third chromosome, with one signal from 9qter retained on the ider(22) and another one moved to a marker chromosome. FISH mapping narrowed the location of the translocation breakpoint to a 1.2 Mb region between RP11-413M3 (at 9q34.3) and the 9q sub-telomeric region. This patient was also found to carry a cryptic deletion of the der(9)t(9;22)(q34;q11) that only affected the 22q11 sequences telomeric to the *ABL1/BCR *fusion junction (3' BCR), while the 9q34 sequences (5' *ABL1*) were retained.

Although none of the other five patients with an isoderivative 22 had a deletion of the derivative 9, a cryptic deletion of *ABL1 *sequences on the other homologue of chromosome 9 [the one not involved in the t(9;22)(q34;q11)] was present in 71% of the metaphases in one patient (no. 13). Both cells with an additional Ph chromosome and cells with an ider(22)t(9;22)(q34;q11) harboured this deletion. The remaining 29% metaphases were also positive for the t(9;22)(q34;q11) but didn't carry the deletion.

Patient no. 19, with a complex variant translocation t(9;22;17;11)(q34;q11.2;q11.2;q13), had five *BCR/ABL1 *fusion positive cell clones, four of them with gains of the 9q34 region, one of which had an ider(22)t(9;22)(q34;q11). In this patient, the cells with an isoderivative Ph chromosome had three copies of the *BCR/ABL1 *fusion and 9q34.12-ter sequences: 1 copy on each arm of the isoderivative Ph chromosome and a third copy on an acrocentric chromosome marker.

### Gains of *BCR/ABL1 *fusion and 9q34 region within marker structures derived from the Ph chromosome

**Figure 3:**
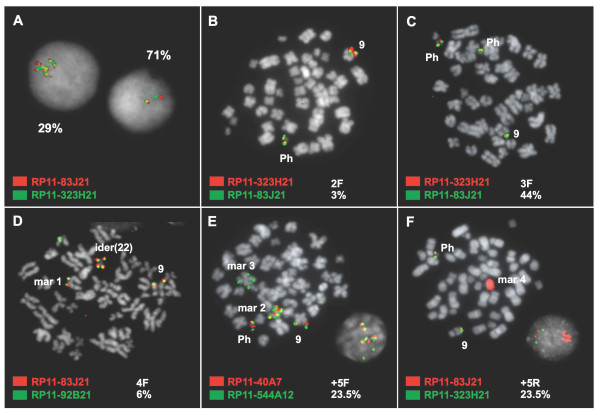
**High copy number gains of *BCR/ABL1 *fusion and downstream region in two CML patients**. **(A) **Representative interphase cells from patient no. 18 with co-hybridization of FISH probes RP11-83J21 (signal in red) and RP11-323H21 (in green). Left, a cluster of fusion signals was detected in 29% of cells. Right, 71% of cells showed only two signals corresponding to both normal homologues of chromosome 9. **(B-F) **Representative metaphase cells from the cell clones found in patient no. 19, indicating the number of fusion signals and the percentage of cells with that signal pattern. 3% of the metaphase cells had one Ph chromosome **(B)**; 44% had two Ph **(C)**; 6% had an isoderivative 22 Ph and a fourth fusion signal on a marker chromosome **(D)**; 23.5% had one Ph, one marker with tandem repeats of the 3'*ABL1*-RAPGEF1 region (1.09 Mb) and one marker with duplication of the 3'*ABL1*-NUP214 region (682 Kb) **(E)**; 23.5% cells had one Ph and one marker with high copy number gains of the 3'*ABL1*-NUP214 region (682 Kb) **(F)**.

Marker structures containing multiple copies of *BCR/ABL1 *fusion and part of the 9q34 region in the form of tandem duplications were found in two patients (no. 18-19) (Figure 3). The size of the amplified region from chromosome 9 was found to be very variable, with a minimum size of the 9q34 amplicon of 682 Kb (no. 19) and a maximum size of 7.23 Mb (no. 18).

Patient no. 18 was found to have an amplification of a large region, from RP11-83J21 at 9q34.1 to RP11-413M3 at 9q34.3. FISH with probes from 9q34 gave a cluster of multiple signals in 62 interphase cells (29%), while the remaining 151 analyzed nuclei lacked the t(9;22)(q34;q11) rearrangement, as evidenced by the two red, two green signal pattern obtained with the use of a *BCR/ABL1 *Dual Color, Dual Fusion Translocation Probe. All BAC probes from the panel used in this study were found to be amplified. However, hybridization with a 9q sub-telomeric probe only gave 1 signal in the cells with amplification of *BCR/ABL1 *fusion, indicating the presence of a breakpoint between RP11-413M3 at 9q43.3 and the sub-telomeric region and thus delimiting the length of the amplicon to 6.03-7.23 Mb. We were only able to detect 43 metaphases, from which 42 were negative for the t(9;22)(q34;q11) rearrangement while only one was positive and contained the amplification. The amplified signal was assigned to a marker chromosome.

On the other hand, during disease progression several subclones stemmed from the original Ph positive clone present in patient no. 19 at diagnosis. Thus, different secondary rearrangements of the Ph chromosome were seen in different subclones showing increasing levels of amplification: 3% of the metaphase cells had one Ph chromosome; 44% had two Ph; 6% had one ider(22)t(9;22)(q34;q11) with one copy of *BCR/ABL1 *fusion and 9q34.1-qter region on each arm, plus another copy of the same region on a chromosome marker (mar1); 23.5% had one Ph, one marker (mar2) with tandem repeats of 3'*ABL1*-RAPGEF1 region (1.09 Mb, from RP11-83J21 to RP11-323H21) and one marker (mar3) with duplication of 3'*ABL1*-NUP214 region (682 Kb, from RP11-83J21 to RP11-643E14) (> 5 copies of 3'*ABL1 *and adjacent downstream sequences); and 23.5% cells had one Ph and one marker (mar4) with high copy number gains of 3'*ABL1*-NUP214 region 682 Kb, from RP11-83J21 to RP11-643E14) (> 5 copies of 3'*ABL1 *and adjacent downstream sequences). The latter chromosome marker contained such a high number of copies of the amplified region that it appeared totally "painted" when using any of the FISH BAC probes contained within the amplified region.

### Gains of *BCR/ABL1 *fusion and downstream region in human CML cell lines

FISH screening of the cell lines for gains of *BCR/ABL1 *fusion and the region downstream of the *ABL1 *breakpoint found the presence of at least two typical Ph chromosomes in five out of the ten cell lines that were analyzed: BV173, JK-1 and KCL-22 with two copies of the Ph chromosome each, LAMA-84 with four copies and EM-2 with either three, four or five copies. Additionally, the Ph negative *BCR/ABL1 *fusion positive cell line CML-T1 with the fusion gene on a masked Ph had three clones, all of them with a duplication of the masked Ph resembling a classical duplication of the Ph chromosome. The remaining cell lines displayed gains that did not correspond to the presence of two identical Ph chromosomes. KU812 and K562 displayed marker chromosomes with high levels of amplification as seen in two patients from this study, while MC3 and MEG-01 harboured duplications within small chromosomes resembling the standard Ph chromosome, a phenomenon that was not detected in any of the patients of this study. None of the ten cell lines was found to have an ider(22)t(9;22)(q34;q11). An overview of the cell lines characteristics and the FISH results is presented in Table 3.

**Table 3:** Metaphase FISH screening in human CML cell lines

Cell line	Source	Cell type	Signal pattern with RP11-83J21(G) and RP11-323H21(R) probes		Location of 9q subtelomeric probe		Location of 22q subtelomeric probe	
BV173	PB	B cell precursor	i.	2F: der(22)×2[20]	i.	der(22)×2	i.	22,der(9)

CML-T1^1^	PB	T cell	i.	3F: 9,der(22)×2	i.	9,der(9)		NT
			ii.	6F: 9×2,der(22)×4	ii.	9×2,der(9)×2		
			iii.	4F: der(22)×4	iii.	der(9)×2		

EM-2	BM	Myeloid	i.	3F: der(22)×3[1]	i.	der(22)×3	i.	22,der(9)×2
			ii.	4F: der(22)×4[20]	ii.	der(22)×4		
			iii.	5F: der(22)×5[21]	iii.	der(22)×5		

JK-1	BST	Myeloid	i.	3F: 9,der(22)×2[23]	i.	9,der(22)×2	i.	22,der(9)

K562	PE	Myeloid	i.	+5F: marker×2, der(9)t(9;17), der(9)t(9;9)[20]	i.	der(9)t(9;17), der(9)t(9;9)	i.	22×2

KCL-22	PE	Myeloid	i.	3F: 9,der(22)×2[20]	i.	9,der(22)×2	i.	22,der(9)

KU812	PB	Myeloid early	i.	2F: der(22)×2[15]	i.	der(22)×2	i.	der(9)×2
		basophilic	ii.	+5F: der(22), marker[9]	ii.	der(22), marker		

LAMA-84	PB	Granulocytic, megakaryocytic and erythroid	i.	4F: der(22)×4[20]	i.	der(22)×4	i.	22,der(9)×2

MC3^2^	PB	Myeloid, lymphoid and megakaryocytoid	i.	2G: der(22)*,der(22)** 2F: 9,der(22)* [19]	i. ii.	9,der(22)* 9,der(22)****	i. ii.	22,der(9) 22,der(9)
			ii.	2G: der(22)***				
				2F: 9,der(22)**** [3]				

MEG-01	BM	Megakaryoblastic	i.	3F: der(22), der(15)t(15;22)[36]	i.	der(7)t(7;22), der(15)t(15;22)	i.	der(9)×2

^1 ^CML-T1 is a Ph negative *BCR/ABL1 *positive cell line. The Ph chromosomes are indeed masked Ph chromosomes with an ins(22;9)(q11.2;q34.1q34.2). CML-T1 had 3 cell clones: one diploid and two tetraploid with/without a deletion of 9q34 on the "normal" 9.^2 ^MC3 had two Ph chromosomes with apparently classical morphology but FISH revealed the presence of two clones both with different Ph chromosome markers carrying cryptic duplications. Stars indicate the different derivative chromosomes 22. Clone i., der(22)* retained all 3' *ABL1 *→ 9qter sequences and carried a duplication of both 9q telomere and a 438 Kb fragment from chromosome 9 (RP11-83J21 → RP11-544A12); der(22)** only retained a 682 Kb fragment from 9 (RP11-83J21 → RP11-643E14). Clone ii., der(22)*** only retained a 438 Kb region from 9 (RP11-83J21 → RP11-544A12) which was duplicated; der(22) ****  retained all 3' *ABL1 *→ 9qter sequences and carried a duplication of 9q telomere.

**Figure 4:**
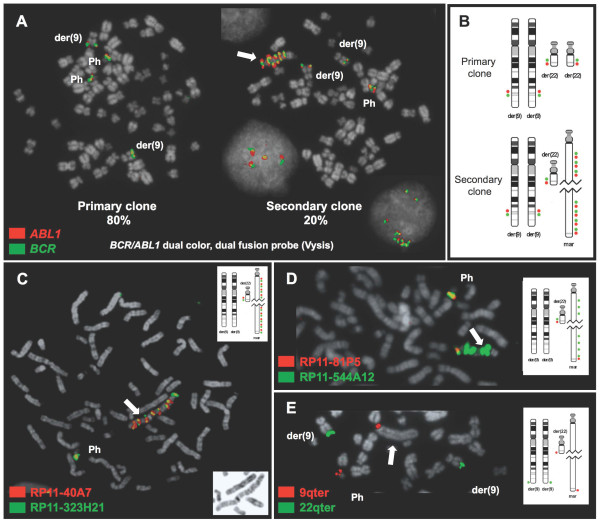
**Gains of *BCR/ABL1 *fusion and a 1.09 Mb region from 9q34 in the cell line KU812**. **(A) **Two representative metaphase cells hybridized with a *BCR/ABL1 *dual colour, dual fusion FISH probe (D-FISH probe; Vysis) showing the primary clone (80% of cells) with an extra Ph chromosome and a secondary clone (20% of cells) with one Ph and and a marker chromosome with tandem duplications of the *BCR/ABL1 *fusion (block arrow). **(B) **Ideograms showing the hybridization signal pattern of the *BCR/ABL1 *D-FISH probe on the primary (top) and secondary (bottom) clones. There are no normal copies of neither chromosome 9 nor chromosome 22. **(C) **Representative metaphase cell from the secondary clone with co-hybridization of RP11-40A7 (signal in red) and RP11-323H21 (5' *RAPGEF1*; in green) showing one fusion signal on a Ph chromosome and tandem duplications of the fusion signal on a marker chromosome (block arrow). Top right, ideogram showing the FISH signal pattern obtained with these probes. Bottom, a close-up of the DAPI banding of the marker chromosome harbouring the tandem duplications. **(D) **Close-up of a representative metaphase co-hybridized with RP11-544A12 (in green) and RP11-81P5 (in red) showing just one signal from the latter on the marker chromosome (block arrow). An ideogram with the hybridization signal patterns of the two probes is shown on the right. The distal breakpoint of the 9q34 amplicon falls between RP11-323H21 and RP11-81P5 and is estimated to measure approx. 1.09 Mb. Sequences downstream of the distal breakpoint of the 9q34 amplicon also hybridized on the marker chromosome but were not amplified. **(E) **Close-up and ideogram of the amplified marker with co-hybridization of 9q and 22q sub-telomeric probes (in red and green, respectively).

The cell line KU812, described to have two Ph chromosomes and two derivative 9 chromosomes, was found to harbour a subclone with one Ph chromosome, two derivatives 9 and a large chromosome marker with homogeneously staining regions containing tandem duplications of *BCR/ABL1 *fusion and part of downstream sequences (Figure 4). The chromosome marker was detected in 20% of the cells and the 9q34 amplicon was measured to be 1.09 Mb long, from RP11-83J21 to RP11-323H21 (9q34.12-q34.13). In addition, the sequences distal to RP11-323H21 (9q34.13-qter) were also present at one end of the marker but only in one copy.

**Figure 5:**
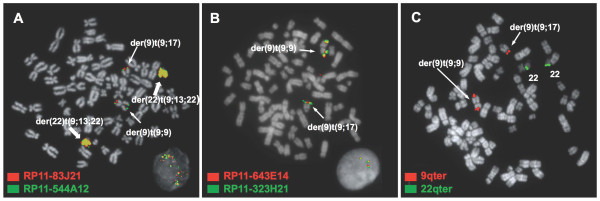
**Gains of *BCR/ABL1 *fusion and a 438 Kb region from 9q34 in the cell line K562**. **(A) **A representative metaphase cell and interphase cell with co-hybridization of FISH probes RP11-83J21 (3' *ABL1*; signal in red) and RP11-544A12 (*NUP214*; in green) showing two acrocentric markers der(22)t(9;13;22)(q34;q11;q11) (indicated by block arrows) with multiple fusion signals, plus two fusion on a der(9)t(9;9) and one fusion signal on a der(9)t(9;17). **(B) **A representative metaphase cell and interphase cell with co-hybridization of FISH probes RP11-643E14 (in red) and RP11-323H21 (5' RAPGEF1; in green) showing just two fusion signals on der(9)t(9;9) and one on der(9)t(9;17), with no hybridization on the markers with *BCR/ABL1 *amplification. The distal breakpoint of the amplicon falls between RP11-544A12 and RP11-643E14 and the amplified region from 9q34 is estimated to be 438 Kb long. **(C) **Representative metaphase cell with co-hybridization of 9q and 22q sub-telomeric FISH probes (in red and green, respectively) showing two signals from 22qter on two chromosomes 22 and three signals from 9qter on both arms of der(9)t(9;9) and on der(9)t(9;17).

On the other hand, the cell line K562 (Figure 5) lacks a classical Ph but it does have two acrocentric markers derived from the Ph chromosome harbouring the *BCR/ABL1 *fusion. These two markers, described by Gribble et al. [10] as der(22)t(9;13;22)(q34;q11;q11), contain high copy number gains of 22q11, *BCR/ABL1 *fusion, 9q34 and 13q31 sequences. The 9q34.12-q34.13 amplicon is covered by three overlapping BAC clones, RP11-83J21, RP11-143H20 and RP11-544A12, measuring 438 Kb. Neither 9q nor 22q telomeres are present in these markers. In addition, K562 has a large chromosome der(9)t(9;9) with the 9q34.12-qter region present in both arms, an acrocentric chromosome der(9)t(9;17)(p?21;p?13) containing a third copy of the 9q34.12-qter region, a small marker chromosome derived from the short arm of chromosome 9, and two apparently normal chromosomes 22.

Furthermore, duplications within apparently typical Ph chromosomes were unmasked in two cell lines. MC3 had two Ph chromosomes with classical morphology, but FISH with BAC probes revealed two clones, both with different Ph chromosome markers containing cryptic duplications. In the main clone, one of the two der(22)t(9;22)(q34;q11) only carried a small fragment (682 Kb) from chromosome 9, from RP11-83J21 to RP11-643E14 (9q34.12-q34.13), and did not carry neither 9q nor 22q telomeres. The other derivative chromosome 22 retained all 3' *ABL1 *→ 9qter sequences and also harboured a cryptic duplication of a 9q34.12-q34.13 fragment from RP11-83J21 to RP11-544A12 (438 Kb long) plus a duplication of the 9q telomere. In contrast, another subset of cells had a Ph chromosome with a typical morphology by G-banding carrying only a 438 Kb region from 9q34 (from RP11-83J21 to RP11-544A12), which was also duplicated. In contrast, a duplication of the 9q telomere region (one in each arm) was present on the other "Ph", which retained all 3' *ABL1 *→ 9qter sequences.

Finally, the cell line MEG-01 also had an apparently typical Ph chromosome harbouring a duplication of *BCR/ABL1 *fusion and 9q34 sequences. However, the duplicated 9q34 fragment was larger, spanning 5.16 Mb from RP11-83J21 to RP11-100C15, while the sequences downstream of RP11-100C15 were missing in the atypical Ph but present on a chromosome identified as der(7)t(7;22). MEG-01 was also found to have an acrocentric chromosome, possibly a der(15)t(15;22)(?p11;q11), containing *BCR/ABL1 *fusion and 9q34.12-qter sequences, in addition to two der(9)t(9;22) chromosomes. It lacked a normal chromosome 22 and a normal 9.

## Discussion

Whereas intrachromosomal amplification of *BCR/ABL1 *is a rare phenomenon, the duplication of the Ph chromosome resulting in two copies of the *BCR/ABL1 *fusion gene is a common abnormality acquired during CML disease progression, although it can be observed during the chronic phase as well. Additional copies of the fusion gene have been described in isochromosomes derived from the Ph chromosome [5,11] and within homogeneously stained regions in marker structures acquired during disease progression, both in patients treated with imatinib [6] or other therapies [7,12]. Using BAC array comparative genome hybridization (CGH) on CML patients and cell lines, Brazma et al. [9] identified gains of 22q11.2 along with gains of *BCR/ABL1 *fusion and only part of the 9q34.1-qter region, therefore not corresponding to the presence of two typical Ph chromosomes.

In our study, we used FISH with a range of BAC probes from 9q34.1-qter on a collection of chromosome suspensions from CML patients and human CML cell lines in order to investigate whether the bone marrow cells with an extra Ph chromosome carry indeed two identical copies of the Ph or, instead, one or both Ph contain cryptic imbalances of some regions. The dual colour FISH assay that we designed and initially tested in all samples contained the BAC clones RP11-83J21 and RP11-323H21; the former targets the 3' end of *ABL1 *gene incorporating the whole of the coding region at 9q34.12 and was thus selected to detect the *BCR/ABL1 *fusion gene, while the latter targets a region 730 Kb downstream of the *ABL1 *gene and is present within the proximal breakpoint cluster region as identified in Ph negative *BCR/ABL1 *positive CML [13]. Each sample was initially tested with this dual FISH probe set to screen for differences between the two Ph chromosomes with emphasis on gains of *ABL1 *and the 9q34.12-q34.13 regions. Cases with an unexpected FISH signal pattern differing from the typical duplication of the Ph chromosome were further characterized using a set of BAC FISH probes from the 9q34.12-qter regions.

A simple duplication of the Ph chromosome with no further events involving the derivative 22 was found in twelve out of nineteen patients and six cell lines. In contrast, a sideline with either one or two isochromosomes of the Ph chromosome was identified in six patients but none of the cell lines. The presence of multiple copies of the isoderivative Ph chromosome is a rare rearrangement that has been previously described [14,15]. In one of our patients a translocation between the distal end of one arm of the isoderivative chromosome 22 and a marker chromosome was revealed. It should be noted than in our study the clone carrying the ider(22)t(9;22)(q34;q11) represents only a small proportion of the *BCR/ABL1 *fusion positive cells (around 5%) in contrast with the cases previously reported, however the clinical significance of the presence in such a small population of cells cannot be defined. One out of the six patients with ider(22)t(9;22)(q34;q11) and a seventh patient were found to carry tandem duplications of the fusion gene and further material from chromosome 9 within marker chromosomes derived from the der(22)t(9;22). We demonstrated the following regions of amplification: 9q34.1 → q34.2 and 9q34.1 → qter, with a common minimum amplified region of 682 Kb. One of the two patients had developed indeed two sidelines with two different marker chromosomes harbouring 9q34 amplicons, apart from the sideline with an isoderivative chromosome 22. Similar amplifications were identified in the imatinib resistant cell line K562, as well as in KU812.

Gadzicki et al. [16] showed that genomic *BCR/ABL1 *fusion amplification resulted in an increase level of *BCR/ABL1 *transcript. However, our study was made with archival stored chromosome suspensions and neither DNA nor RNA material from these samples was available to investigate the expression levels. Morel et al. [17] also reported the amplification of *BCR/ABL1 *in multiple double minutes (3-30) in a CML patient with imatinib resistance, but none of the patients from the present study contained such structures. Instead, the intrachromosomal amplification of *BCR/ABL1 *fusion seen in chromosome markers in our two patients is very similar to amplification of the fusion gene in each of the two *BCR/ABL1 *fusion positive markers of the cell line K562, although the distal breakpoints of the amplicon are different. We detected the presence of a subclonal cell population in two cell lines, KU812 and MC3. Even though they may be an *in vitro *artifact resulting from repeated passaging, it is interesting that in KU812 the acrocentric chromosome marker that harbours tandem duplications of *BCR/ABL1 *fusion and a 1 Mb long 9q34 amplicon is similar to the chromosome markers that some CML patients acquire *in vivo*, suggesting the possibility that this subclone had already been present in the original sample when the cell line was established and had expanded under *in vitro *cell culture conditions thanks to a proliferative advantage.

## Conclusions

In summary, 58% of the patients and 50% of the cell lines were found to harbour two identical Ph chromosomes, while the remaining cases had isoderivative chromosomes 22 or gains of *BCR/ABL1 *fusion gene accompanied by 9q34 material harboured by marker chromosomes. Taken together, these data confirm that the genomic amplification of *BCR/ABL1 *fusion that occurs in some CML patients during disease progression also involves amplification of 9q34 gene-rich sequences downstream of *ABL1 *breakpoint. Moreover, the variety of rearrangements and breakpoints identified in this relatively small cohort of CML patients' samples shows that the Ph chromosome is not a stable structure but prone to further rearrangements during disease progression.

## Methods

### Patients

Archival fixed chromosome suspensions (stored at -20°C) from the bone marrow of nineteen CML patients with either more than 1 Ph detected by G-banding or more than one copy of *BCR/ABL1 *fusion detected by FISH were donated anonymously for further analysis by the Hammersmith Hospital, the University College Hospital and the Royal Free Hospital (London, UK); and St Anna Hospital (Sofia, Bulgaria). The samples were originally taken between the years 1998 and 2008 for the detection of the t(9;22)(q34;q11). Eleven patients carried a typical t(9;22)(q34;q11) and three patients carried variant translocations. Neither the karyotype nor the FISH results were available for the remaining five samples. In addition, multiple copies of *BCR/ABL1 *fusion had been detected by interphase FISH in one patient.

### Cell lines

Ten human CML cell lines (BV173, CML-T1, EM-2, JK-1, K562, KCL-22, KU812, LAMA-84, MC3 and MEG-01) were obtained from the German Collection of Microorganisms and Cell Cultures/Department of Human and Animal Cell Cultures (DSMZ, Braunschweig, Germany). Cells were cultured in RPMI 1640 medium (Invitrogen, Paisley, UK) supplemented with 10-20% (v/v) foetal bovine serum (Invitrogen, Paisley, UK), 1% (v/v) L-glutamin (Invitrogen, Paisley, UK) and 1% (v/v) penicillin/streptomycin (Invitrogen, Paisley, UK) at 37°C in 5% CO2. Cells were blocked at the metaphase stage by treatment with Colcemid (Invitrogen, Paisley, UK) and chromosomes harvested by means of a hypotonic treatment (0.075 M KCl) followed by fixation with methanol-acetic acid (3:1).

### Fluorescence *in situ *hybridization (FISH)

FISH studies were performed on interphase and metaphase cells using a panel of BAC probes from 9q34.1-qter. BAC clones were selected according to the UCSC genome browser (University of California Santa Cruz, CA, USA), assembly NCBI36/hg18, and obtained from the BACPAC Resources Center (Children's Hospital Oakland Research Institute, Oakland, CA, USA), the Sanger Centre (Cambridge, UK) and Invitrogen (Paisley, UK). After growth in LB medium (Sigma-Aldrich, Dorset, UK) with chloramphenicol, the DNA was extracted with a QIAGEN Large-Construct Kit (Qiagen, West Sussex, UK), directly labelled with either Spectrum Orange or Spectrum Green dUTPs (Vysis Inc., Downers Grove, IL, USA) with a Nick Translation Kit (Vysis Inc., Downers Grove, IL, USA), precipitated with ethanol and resuspended in hybridization buffer composed of 50% formamide, 10% dextran sulphate (Sigma-Aldrich, Dorset, UK), 0.1% Tween 20 (Sigma-Aldrich, Dorset, UK) and 10 mM Tris in 2 × SSC. Each clone was first tested on normal human metaphase cells in order to verify the mapping location. All tests were carried out as dual colour, dual locus FISH experiments. Commercial sub-telomeric FISH probes from 9q and 22q (Stretton Scientific Ltd, Stretton, UK) were used according to the manufacturer's instructions to identify the location of the telomeres, while FISH with the commercially available LSI *BCR/ABL1 *Dual Color, Dual Fusion Translocation Probe (Vysis Inc., Downers Grove, IL, USA) was performed on the cell lines following the manufacturer's protocol.

Fresh slides were made from chromosome suspensions and aged overnight at room temperature. Briefly, the slides were treated with RNase A (100 μg/ml in 2 × SSC; 1 hour, 37°C), digested with pepsin (0.025% in 0.01 N HCl; 8 minutes, 37°C), dehydrated in a graded ethanol series (70%-90%-100%; 2 minutes each), and denatured in 70% formamide/2 × SSC (2 minutes; 72°C) followed by dehydration in ice-cold ethanol series. Denatured probes (73°C, 5 minutes) were applied to the slides. After overnight hybridization at 37°C, the slides were washed in 0.4× SSC/0.3% IGEPAL (73°C, 5 minutes) and two times in 2 × SSC/0.1% IGEPAL (37°C, 5 minutes). The cells were counterstained with 4,6-diamidino-2-phenylindole (Vysis Inc., Downers Grove, IL, USA) in Vectashield mounting medium (Vector Laboratories, Burlingame, CA, USA) for chromosome visualization. FISH analysis was carried out using an automated epifluorescence microscope (BX61, Olympus UK Ltd, Essex, UK) equipped with a CCD camera (ORCA_II_-ER, Hammamatsu Photonics UK Ltd., Hertfordshire, UK). Digital images were captured using SmartCapture 3 software (Digital Scientific Ltd., Cambridge, UK).

## Competing interests

The authors declare that they have no competing interests.

## Authors' contributions

AV carried the FISH studies and drafted the manuscript. EPN conceived and designed the study, and helped to draft the manuscript. All authors read and approved the final manuscript.

## Supplementary Material

Additional file 1 **FISH mapping carried out on the CML patient showing for each case the number of signals obtained with each probe and their location**. Click here for file
