# Supplementary material for: Genomic amplification of BCR/ABL1 and a region downstream of ABL1 in chronic myeloid leukaemia: a FISH mapping study of CML patients and cell lines
Source: Mol Cytogenet. 2010 Sep 1;3:15. doi: 10.1186/1755-8166-3-15 (PMC2944125; doi:10.1186/1755-8166-3-15)
Supplement: Additional file 1 — FISH mapping carried out on the CML patient showing for each case the number of signals obtained with each probe and their location. [file 1755-8166-3-15-S1.DOC]

| **ID** | **RP11-83J21** | **RP11-143H20** | **RP11-544A12** | **RP11-643E14** | **RP11-40A7** | **RP11-323H21** | **RP11-81P5** | **RP11-92B21** | **RP11-413M3** | **9qter** | **22qter** |
| --- | --- | --- | --- | --- | --- | --- | --- | --- | --- | --- | --- |
| 1 | 2:9,Ph/  3:9,2xPh | NT | NT | NT | NT | 2:9,Ph/  3:9,2xPh | NT | NT | NT | NA | NA |
| 2 | 2:9,Ph/  3:9,2xPh | NT | NT | NT | NT | 2:9,Ph/  3:9,2xPh | NT | NT | NT | 2:9,Ph/  3:9,2xPh | 2:22,der(9) |
| 3 | 3:9,2xPh | NT | NT | NT | NT | 3:9,2xPh | NT | NT | NT | 3:9,2xPh | 2:22,der(9) |
| 4 | 3:9,2xPh | NT | NT | NT | NT | 3:9,2xPh | NT | NT | NT | 3:9,2xPh | 2:22,der(9) |
| 5 | 3:9,2xPh | NT | NT | NT | NT | 3:9,2xPh | NT | NT | NT | 3:9,2xPh | 2:22,der(9) |
| 6 | 2:9,Ph/  3:9,2xPh | NT | NT | NT | NT | 2:9,Ph/  3:9,2xPh | NT | NT | NT | NA | NA |
| 7 | 2:9,Ph/  3:9,2xPh | NT | NT | NT | NT | 2:9,Ph/  3:9,2xPh | NT | NT | NT | NA | NA |
| 8 | 2:2x9/  2:2xPh | NT | NT | NT | NT | 2:2x9/  2:2xPh | NT | NT | NT | 2:2x9/  2:2xPh | 2:2x22/  2:22,der(9) |
| 9 | 2:9,Ph/  3:9,2xPh | NT | NT | NT | NT | 2:9,Ph/  3:9,2xPh | NT | NT | NT | 2:9,Ph/  3:9,2xPh | 2:22,der(17) |
| 10 | 2:2x9/  2:9,Ph/  3:9,2xPh | NT | NT | NT | NT | 2:2x9/  2:9,Ph/  3:9,2xPh | NT | NT | NT | 2:2x9/  2:9,Ph/  3:9,2xPh | 2:2x22/  2:22,der(?19) |
| 11 | 2:9,der(1)/  3:9,2xder(1) | NT | NT | NT | NT | 2:9,der(1)/  3:9,2xder(1) | NT | NT | NT | 2:9,der(1)/  3:9,2xder(1) | 2:mar, der(9) |
| 12 | 2:9,Ph/  3:9,2xPh | NT | NT | NT | NT | 2:9,Ph/  3:9,2xPh | NT | NT | NT | NT | NT |
| 13 | 2:9,Ph/  1:Ph/  2:2xPh/  **2:ider(22)** | 2:9,Ph/  1:Ph/  2:2xPh/  **2:ider(22)** | NT | 2:9,Ph/  3:9,2xPh/  **3:9,ider(22)** | NT | 2:9,Ph/  3:9,2xPh/  **3:9,ider(22)** | NT | 2:9,Ph/  3:9,2xPh  **3:9,ider(22)** | NT | 2:9,Ph/  3:9,2xPh/  **3:9,ider(22)** | NT |
| 14 | 2:2x9/  2:9,Ph/  **3:9,ider(22)** | NT | 2:2x9/  2:9,Ph/  **3:9,ider(22)** | NT | 2:2x9/  2:9,Ph/  **3:9,ider(22)** | 2:2x9/  2:9,Ph/  **3:9,ider(22)** | 2:2x9/  2:9,Ph/  **3:9,ider(22)** | 2:2x9/  2:9,Ph/  **3:9,ider(22)** | 2:2x9/  2:9,Ph/  **3:9,ider(22)** | 2:2x9/  2:9,Ph/  **3:9,ider(22)** | 2:2x22/  2:22,der(9) |
| 15 | 2:9,Ph/  3:9,2xPh/  **3:9,ider(22)** | NT | 2:9,Ph/  3:9,2xPh/  **3:9,ider(22)** | NT | NT | 2:9,Ph/  3:9,2xPh/  **3:9,ider(22)** | 2:9,Ph/  3:9,2xPh/  **3:9,ider(22)** | NT | NT | 2:9,Ph/  3:9,2xPh/  **3:9,ider(22)** | 2:22,der(9) |
| 16 | 2:9,Ph/  3:9,2xPh/  **5:9,2xider(22)** | NT | NT | NT | NT | 2:9,Ph/  3:9,2xPh/  **5:9,2xider(22)** | NT | NT | NT | 2:9,Ph/  3:9,2xPh/  **5:9,2xider(22)** | NT |
| 17 | 2:2x9/  2:9,Ph/  3:9,2xPh/  **3:9,ider(22)** | NT | 2:2x9/  2:9,Ph/  3:9,2xPh/  **3:9,ider(22)** | NT | 2:2x9/  2:9,Ph/  3:9,2xPh/  **3:9,ider(22)** | 2:2x9/  2:9,Ph/  3:9,2xPh/  **3:9,ider(22)** | 2:2x9/  2:9,Ph/  3:9,2xPh/  **3:9,ider(22)** | NT | 2:2x9/  2:9,Ph/  3:9,2xPh/  **3:9,ider(22)** | 2:2x9/  2:9,Ph/  3:9,2xPh/  **3:9,ider(22), mar ^1^** | 2:2x22/  2:22,der(9) |
| 18 | 2:2x9/  **+3F:9,mar(HCN)** | NT | NT | 2:2x9/  **+3F:9,mar(HCN)** | 2:2x9/  **+3F:9,mar(HCN)** | 2:2x9/  **+3F:9,mar(HCN)** | 2:2x9/  **+3F:9,mar(HCN)** | 2:2x9/  **+3F:9,mar(HCN)** | 2:2x9/  **+3F:9,mar(HCN)** | 2:2x9/  **1:9** | NT |
| 19 | 2:9,Ph/  3:9,2xPh/  **4:9,ider(22),mar**/  **+3F:9,Ph,mar1(HCN)**/  **+3F:9,Ph,mar,mar2(HCN)** | NT | 2:9,Ph/  3:9,2xPh/  **4:9,ider(22),mar**/  **+3F:9,Ph,mar1(HCN)**/  **+3F:9,Ph,mar,mar2(HCN)** | 2:9,Ph/  3:9,2xPh/  **4:9,ider(22),mar**/  **+3F:9,Ph,mar1(HCN)**/  **+3F:9,Ph,mar,mar2(HCN)** | 2:9,Ph/  3:9,2xPh/  **4:9,ider(22),mar**/  **+3F:9,Ph,mar2(HCN)** | 2:9,Ph/  3:9,2xPh/  **4:9,ider(22),mar**/  **+3F:9,Ph,mar2(HCN)** | 2:9,Ph/  3:9,2xPh/  **4:9,ider(22),mar** | 2:9,Ph/  3:9,2xPh/  **4:9,ider(22),mar** | 2:9,Ph/  3:9,2xPh/  **4:9,ider(22),mar** | 2:9,Ph/  3:9,2xPh/  4:9,ider(22),mar | 2:22,?der(17) |

^1^Just one copy on the ider(22)

Abbreviations: +3F, more than three fusion signals; HCN, high copy numbers; mar, chromosome marker; NT, not tested
